# Alterations in Ileal Microbiota and Fecal Metabolite Profiles of Chickens with Immunity to *Eimeria mitis*

**DOI:** 10.3390/ani14233515

**Published:** 2024-12-05

**Authors:** Zhongchuang Wang, Peiyao Shang, Xingju Song, Minghui Wu, Tong Zhang, Qiping Zhao, Shunhai Zhu, Yu Qiao, Fanghe Zhao, Ruiting Zhang, Jinwen Wang, Yu Yu, Hongyu Han, Hui Dong

**Affiliations:** 1 Key Laboratory of Animal Parasitology of Ministry of Agriculture, Shanghai Veterinary Research Institute, Chinese Academy of Agricultural Sciences, Minhang, Shanghai 200241, China; zxclkjhf1999601@163.com (Z.W.); zqp@shvri.ac.cn (Q.Z.); zhushunhai@shvri.ac.cn (S.Z.); hhysh@shvri.ac.cn (H.H.); 2 College of Animal Science and Technology, Guangxi University, Nanning 530004, China; songxingju@gxu.edu.cn; 3 Beijing Yuanda Spark Medical Technology Co., Ltd., Beijing 102615, China

**Keywords:** Coccidia, *Eimeria mitis*, gut microbiota, fecal metabolites

## Abstract

Coccidiosis, caused by different species of *Eimeria* parasites, is an economically important disease in poultry and livestock worldwide. This study aimed to investigate the changes in the ileal microbiota and fecal metabolites in chickens after repeated infections with low-dose *E. mitis*. The chickens developed solid immunity against a high dose of *E. mitis* infection after repeated infections with low-dose *E. mitis*. The composition of the ileal microbiota and the metabonomics of the *Eimeria*-immunized group and the control group were detected using 16S rRNA sequencing and liquid chromatography–mass spectrometry (LC-MS). The relative abundance of *Neisseria*, *Erysipelotrichaceae*, *Incertae sedis*, *Coprobacter*, *Capnocytophaga*, *Bifidobacterium*, and the *Ruminococcus torques* group declined in the *Eimeria*-immunized chickens, whereas *Alloprevotella*, *Staphylococcus*, *Haemophilus*, and *Streptococcus* increased. Furthermore, 286 differential metabolites (including N-undecylbenzenesulfonic acid, 1,25-dihydroxyvitamin D3, gluconic acid, isoleucylproline, proline, and 1-kestose) and 19 significantly altered metabolic pathways (including galactose metabolism, ABC transporters, starch and sucrose metabolism, the ErbB signaling pathway, and the MAPK signaling pathway) were identified between the *Eimeria*-immunized group and the control group. These discoveries will help us learn more about the composition and dynamics of the gut microbiota as well as the metabolic changes in chickens infected with *Eimeria* spp.

## Simple Summary

This study is the first to investigate the effects of *E. mitis* on the ileal microbiota and fecal metabolites of chickens. Non-targeted metabolic technology and 16S rRNA sequencing technology were used to study changes in the ileal microbiota and fecal metabolites after repeated infections with *E. mitis*. The results indicated that chickens developed solid immunity against a high dose of *E. mitis* infection after repeated infections with low-dose *E. mitis* and that repeated low-dose infections of *E. mitis* disrupted the ileal microbiota balance.

## 1. Introduction

Avian coccidiosis is an intestinal disease caused by infection with *Eimeria* spp. It causes severe diarrhea, weight loss, poor nutrient absorption, and increased susceptibility to other pathogens [1]. The global cost of poultry coccidiosis is estimated to exceed USD 14 billion each year, resulting in economic losses through increased drug costs [2]. Seven main species of *Eimeria (E. acervulina*, *E. brunetti*, *E. maxima*, *E. mitis*, *E. necatrix*, *E. praecox*, and *E. tenella*) are recognized worldwide in poultry and vary by geographic region and poultry production system [3]. These *Eimeria* species differ in terms of their invasion of distinct sites within the intestine, pathogenicity, and type of lesion they produce.

The gut, which contains trillions of microorganisms, plays a key role in digestion, the absorption of nutrients, and the regulation of physiological processes, including immune responses and pathogen elimination [4,5,6]. Coccidiosis infection alters the intestinal microbiota’s composition and integrity, resulting in elevated susceptibility to diseases that threaten the overall health and productivity of chickens [7,8,9,10]. It was reported that *E. tenella* infection altered the composition and diversity of cecal microbiota, significantly reducing Proteobacteria and Firmicutes (*Enterococcus*) [11]. In another study, the abundance of *Bacteroides* and *SMB53*, as well as an unclassified genus in the family of *Coriobacteriaceae*, was significantly reduced by *E. maxima* infection [12]. A recently published study showed that infection with mixed *Eimeria* spp. caused a greater abundance of *Clostridium sordellii*, *Clostridium butyricum*, unclassified *Ruminococcaceae*, and unclassified *Bacteroides* in the ileum and a higher abundance of *Clostridium perfringens* and unclassified *Enterobacteriaceae* in the cecum [13].

Metabolomics facilitates a comprehensive examination of metabolic alterations in reaction to external stimuli or disturbances and provides new understandings about disease pathogenesis and infection responses [14,15]. In an experiment with mice infected with *Trypanosoma crudii*, the authors found reductions in glucose import, reductions in fatty acid and phospholipid synthesis in plasma, and an increase in heart tissue [16]. In previous reports, a total of 389 significant metabolites were identified in the liver of acutely infected mice with type II *T. gondii* by using metabolomics, while 368 metabolites were identified in the liver of chronically infected mice compared to the control group [17]. It was reported that after rabbits were infected with *E. magna*, 13 metabolites were changed and 7 metabolic pathways were dysregulated [18]. Another similar study demonstrated that after rabbits were infected with *E. intestinalis*, 20 metabolites and 2 metabolic pathways were altered, with lipid metabolism as the major disrupted metabolic pathway [19].

At present, the changes in the gut microbiota and metabolites caused by *Eimeria* spp. infection are mainly studied under the condition of severe gut damage caused by high-dose oocysts. However, until now, no study has been conducted on the gut microbiota and metabolites of chickens with strong immunity to *Eimeria* spp. *E. mitis* is less pathogenic and parasitizes in the small intestine, and its negative influence is often disregarded in the poultry industry [20,21]. There has been no study on the effects of *E. mitis* on the gut microbiome and metabolites of chickens. In the present study, the chickens were immunized four times with low-dose *E. mitis*, resulting in complete immunity against *E. mitis* infection. Then, the changes in the gut microbiota and metabolites of chickens were analyzed by 16S rRNA sequencing technology and non-targeted metabolic technology, respectively. This study will provide a new insight into the composition and dynamics of the gut microbiota as well as the metabolic changes in chickens infected with *Eimeria* spp.

## 2. Materials and Methods

### 2.1. Parasites and Experimental Chickens

*E. mitis* sporulated oocysts were preserved by the Shanghai Veterinary Research Institute, Chinese Academy of Agricultural Sciences. The oocysts were acquired, purified, and sporulated following established protocols [22]. One-day-old Sanhuang chickens, purchased from Shanghai Minyou Breeding Cooperative, were raised in isolators and maintained at 34–35 °C in a standard animal house without coccidia.

### 2.2. Infection of Chickens

An *Eimeria*-immunized group of 12 chickens was set up, along with a healthy control group of 12 chickens. All chickens freely consumed a non-antibiotic commercial feed. The environment temperature was maintained at 25–30 °C. Seven-day-old chickens were infected using oral gavage with low-dose *E. mitis* (2 × 10^4^) sporulated oocysts (*Eimeria*-immunized group). The uninfected chickens received an equal volume of PBS (control group). A total of four infections were performed throughout the course at 7-day intervals. On the sixth to eighth days after each infection, feces were collected daily from each group for oocysts per gram of feces (OPGs) using McMaster’s counting technique.

### 2.3. Evaluation of Immunization Effectiveness

On the seventh day after the final infection, six chickens from the *Eimeria*-immunized group and six chickens from the control group were selected and challenged with a high dose of *E. mitis* (2 × 10^5^) sporulated oocysts. Fecal oocyst numbers for each group were determined on days 6−7 post-challenge. The remaining *Eimeria*-immunized group (*n* = 6) and the control group (*n* = 6) were kept for subsequent analyses of the gut microbiota and metabolite profiles.

### 2.4. The 16S rDNA Amplification and Bioinformatic Analysis

The fresh contents of the ileum from the remaining chickens were collected, and the total DNA was isolated using a Hipure Soil DNA Kit (Magen, Guangzhou, China) following the manufacturer’s instructions. DNA concentration and integrity were measured using a NanoDrop 2000 (Thermo Fisher Scientific, Waltham, MA, USA) and agarose gel electrophoresis. For bacterial diversity analysis, 16S rRNA genes were amplified with the universal primers 343F (5′-TACGGRAGGCAGCAG-3′) and 798R (5′-AGGGTATCTAATCCT-3′) for the V3-V4 region [23]. After purifying the PCR products with AMPure XP beads, they underwent another round of PCR. The final amplicon was then quantified using the Qubit dsDNA Assay Kit (Thermo Fisher Scientific, Waltham, MA, USA). The concentrations were modified prior to sequencing and subsequently processed using an Illumina NovaSeq 6000 system by OE Biotech Co., Ltd. (Shanghai, China).

Raw sequencing data were in FASTQ format. Paired-end reads were then preprocessed using Cutadapt software to detect and cut off the adapter. After trimming, paired-end reads were filtered with low-quality sequences, denoised, and merged, and the chimera reads were detected and cut off using DADA2 with the default parameters of QIIME2 [24]. At last, the software output the representative reads and the ASV abundance table. The representative read of each Amplicon Sequence Variant (ASV) was selected using the QIIME2 package. All representative reads were annotated and blasted against the Silva database using the q2-feature-classifier with the default parameters.

QIIME2 software was used for alpha and beta diversity analysis. The unweighted UniFrac distance matrix performed by the R package was used for an unweighted UniFrac principal coordinate analysis (PCoA) to estimate the beta diversity [25]. To test for significance in UniFrac distances, the nonparametric permutational analysis of variance (PERMANOVA) test was used. The Wilcoxon statistical test was conducted using the R package to assess significant differences between the *Eimeria*-immunized and the control group. The research utilized the linear discriminant analysis effect size (LEfSe) method to analyze significant biomarker taxa [26].

### 2.5. LC-MS Sample Preparation and Bioinformatic Analysis

Fresh feces from the remaining chickens were collected and snap-frozen in liquid nitrogen. Frozen samples were thawed slowly at room temperature. The extraction and analysis of samples were performed according to previously described methods [27]. The obtained supernatants were filtered using 0.22 μm microfilters and analyzed by LC-MS (OE Biotech Company, Shanghai, China). The samples were subjected to analysis using the Agilent 7890B gas chromatography system and the Agilent 5977B MSD system, according to the established parameters as previously outlined [28].

The raw data were transferred to the analysis base file (ABF) format via the Analysis Base File Converter software. An Orthogonal Partial Least Squares-Discriminant Analysis (OPLS-DA) was used to evaluate the different metabolites between the *Eimeria*-immunized group and the control group. In order to evaluate the quality of the OPLS-DA and avoid overfitting, we utilized a 7-fold cross-validation and 200 response permutation testing (RPT). Variable Importance in the Projection (VIP) obtained from the OPLS-DA model was utilized to assess the contribution of each metabolite to the classification. A two-tailed Student’s t-test was employed to assess the significance of metabolite differences between the *Eimeria*-immunized group and the control group. The selection of important metabolites was chosen based on VIP values > 1.0 and *p*-values < 0.05. Furthermore, we utilized the KEGG database for a comprehensive analysis of the metabolic pathways associated with the identified metabolites.

### 2.6. Statistical Analysis

Data are presented as the mean ± standard error of the mean (SEM). A Student’s *t*-test was performed to determine the significance between the two groups for OPGs; *p* < 0.05 was considered significant.

## 3. Results

### 3.1. Fecal OPG Counts During Immunizations

A higher OPG count was produced on days 6 to 8 after the first infection and relatively low OPGs on the sixth day after the second infection (Table 1). No oocysts were detected from the seventh day after the second infection to the eighth day after the fourth infection.

### 3.2. Immunization Effectiveness

After four infections, the chickens were challenged with a high dose of *E. mitis*. The OPG results are shown in Table 2. Our results demonstrated that the *Eimeria*-immunized group had no oocysts discharged 6–7 days after the challenge. However, the control group had a large number of oocysts discharged. These results indicated that the chickens had developed immunity against a high dose of *E. mitis* infection after repeated infections with low-dose *E. mitis*.

### 3.3. E. mitis Changed Ileal Microbiota Diversity

**Figure 1:**
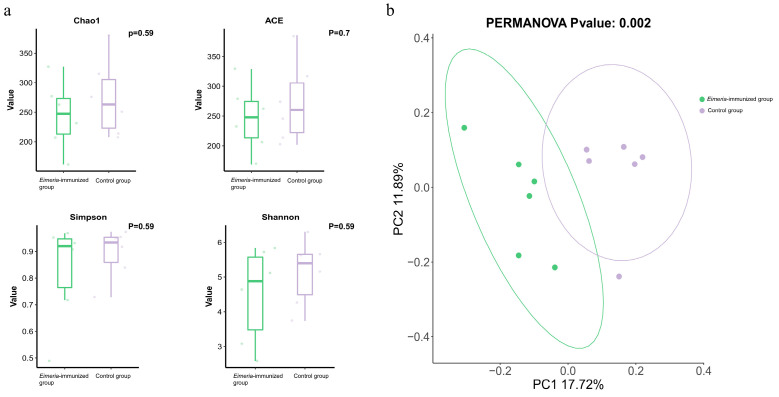
Diversity analysis of the gut microbiota: (**a**) analysis of the alpha diversity between the *Eimeria*-immunized group and the control group; (**b**) PCoA analysis based on the unweighted UniFrac distance.

We assessed the alpha diversity using the Chao1 and Abundance-based Coverage Estimator (ACE) indices to measure microbiota richness and the Shannon and Simpson indices for species diversity. The analysis showed no significant variation in the four indices between individuals within the *Eimeria*-immunized group and the control group (Figure 1a). The evaluation of beta diversity was conducted using an unweighted UniFrac distance analysis. The PCoA result showed that there were significant differences (*p* < 0.05) between the *Eimeria*-immunized group and the control group (Figure 1b).

### 3.4. E. mitis Altered Ileal Microbiota Composition

**Figure 2:**
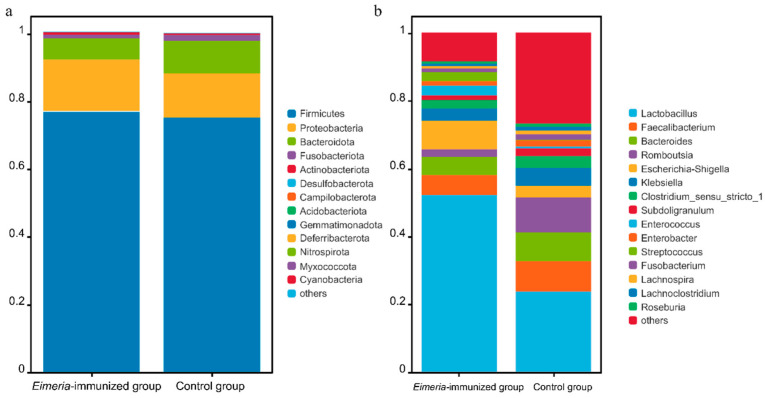
The dominant abundant bacterial phyla and genera are shown. Other phyla and genera were all assigned as “Others”. Gut bacterial composition at the phylum (**a**) and genus (**b**) levels in different experiment groups.

In accordance with Figure 2, our analysis displayed the predominant bacterial phyla and genera based on their relative abundance. The dominant phyla included Firmicutes, Proteobacteria, and Bacteroidetes. In the control group, the relative abundance of Firmicutes, Proteobacteria, and Bacteroidetes was 76.09%, 12.47%, and 9.17%, respectively. In the *Eimeria*-immunized group, the relative abundance of Firmicutes, Proteobacteria, and Bacteroidetes was 77.48%, 14.99%, and 5.71%, respectively. We further compared the bacterial composition of all experimental treatments at the genus level. In the control group, the relative abundance of *Lactobacillus*, *Romboutsia*, *Faecalibacterium*, and *Bacteroides* was 23.61%, 12.41%, 8.46%, and 8.01%, respectively. In the *Eimeria*-immunized group, the relative abundance of *Lactobacillus*, *Romboutsia*, *Faecalibacterium*, and *Bacteroides* was 55.11%, 2.22%, 5.30%, and 4.84%, respectively.

**Figure 3:**
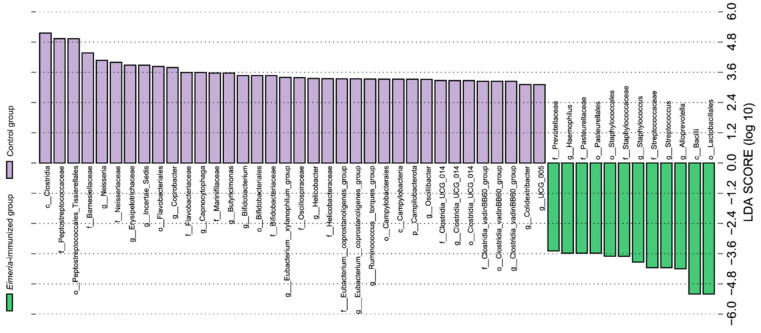
The linear discriminant analysis (LDA) effect size (LEfSe) analysis. Histogram of the linear discriminant analysis (LDA) scores.

We detected significant taxonomic variations between the *Eimeria*-immunized group and the control group by linear discriminant analysis (LDA) scores above 3.0 (Figure 3). At the genus level, the control group displayed a significant increase in the abundance of *Neisseria*, *Erysipelotrichaceae*, *Incertae sedis*, *Coprobacter*, *Capnocytophaga*, *Butyricimonas*, *Bifidobacterium*, the *Eubacterium xylanophilum* group, *Helicobacter*, the *Eubacterium coprostanoligenes* group, the *Ruminococcus torques* group, *Oscillibacter*, *Clostridia UCG-014*, the *Clostridia vadin BB60* group, *Colidextribacter*, and *UCG-005* compared to the *Eimeria*-immunized group. The *Eimeria*-immunized group displayed a significant increase in the abundance of *Alloprevotella*, *Streptococcus*, *Staphylococcus*, and *Haemophilus* compared to the control group.

### 3.5. E. mitis Perturbed Fecal Metabolites

**Figure 4:**
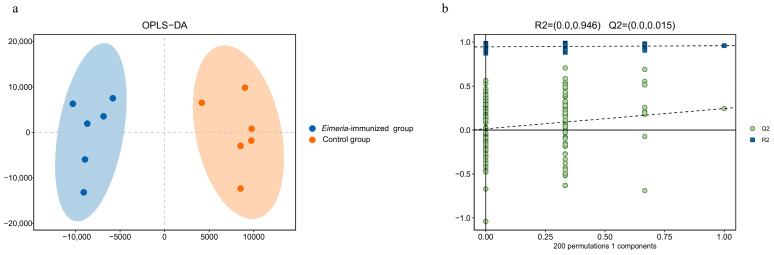
OPLS-DA score scatter plot of the *Eimeria*-immunized group vs. the control group (**a**) and permutation test of the *Eimeria*-immunized group and the control group (**b**).

In order to determine the impact of *E. mitis* on the chickens’ metabolites, an LC-MS untargeted metabolomics analysis was performed. The OPLS-DA score scatter plots showed that the *Eimeria*-immunized group and the control group clearly separated into distinct clusters according to their metabolic differences (Figure 4a). The permutation test was applied to validate the fit accuracy and predictability of the OPLS-DA model (Figure 4b). Between the *Eimeria*-immunized group and the control group, the model’s parameters were R2Y(cum) = 0.959 and Q2(cum) = 0.244. Generally, R2Y(cum) > 0.5 is considered to be good, indicating that the OPLS-DA model does not exceed the fitting.

**Figure 5:**
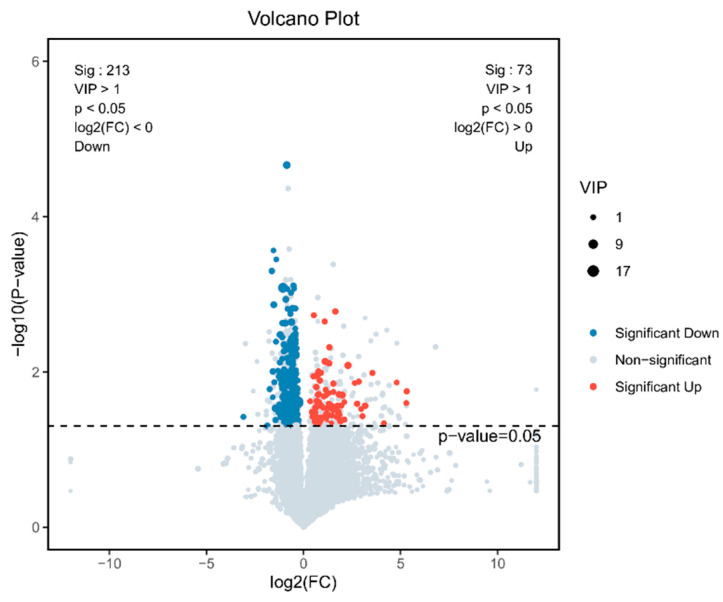
Volcano map of different metabolites: blue represents the downregulated differential metabolites, red represents the upregulated differential metabolites, and gray represents metabolites without differences.

**Figure 6:**
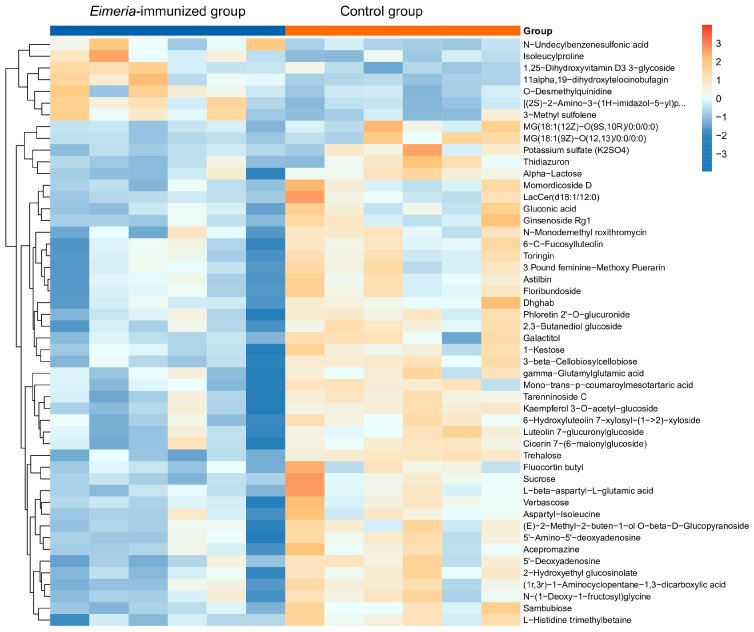
Cluster analysis of the top 50 differential metabolites: each row in the figure represents a differential metabolite, and each column represents a sample.

The difference in screening results shown by the volcano maps (Figure 5) illustrated 286 differential metabolites, 73 of which were upregulated and 213 downregulated in the *Eimeria*-immunized group compared to the control group. A heat map was drawn using the top 50 differential metabolites between the *Eimeria*-immunized group and the control group (Figure 6). Metabolites such as trehalose, gluconic acid, 1-kestose, and ginsenoside Rg1 were significantly reduced in the *Eimeria*-immunized group compared to the control group. Meanwhile, metabolites such as N-undecylbenzenesulfonic acid, 1,25-dihydroxyvitamin D3, and isoleucylproline were significantly increased in the *Eimeria*-immunized group compared to the control group.

**Figure 7:**
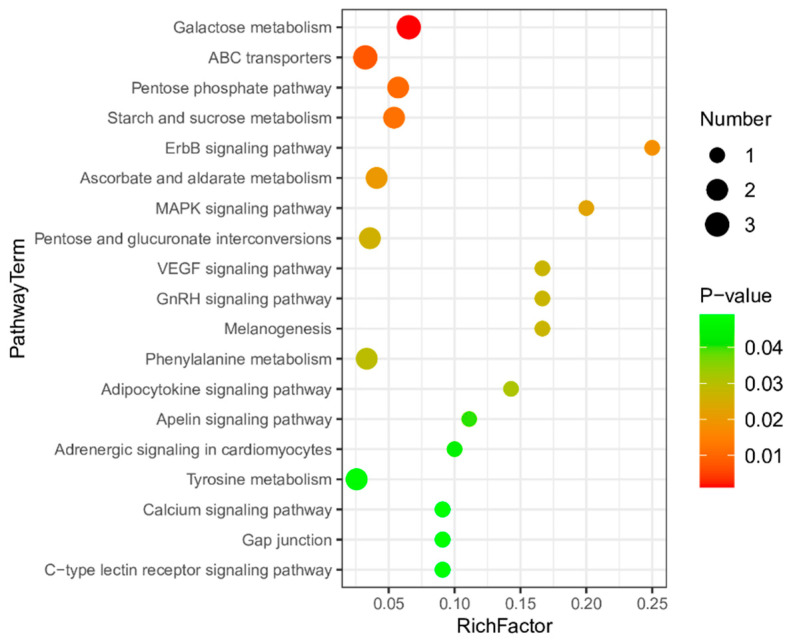
Differentially altered metabolic pathways, as visualized using bubble plots (*p* < 0.05); the bubble size represents the number of metabolites. Coloring from green to red indicates a decrease in the *p*-value; the larger the point, the more metabolites are enriched in the pathway.

Furthermore, we conducted classification and annotation of the detected differential metabolites utilizing the KEGG database. We identified 19 significantly altered metabolic pathways (*p* < 0.05), including galactose metabolism, ABC transporters, starch and sucrose metabolism, the ErbB signaling pathway, the MAPK signaling pathway, the GnRH signaling pathway, and the adipocytokine signaling pathway (Figure 7).

## 4. Discussion

In this experiment, chickens were repeatedly infected with a low dose of *E. mitis*. After four infections, the chickens were challenged with a high dose of *E. mitis*. No oocysts were excreted in their stool, indicating the chickens had immunity to *E. mitis* infection. Non-targeted metabolic technology and 16S rRNA sequencing technology were used to study changes in the ileal microbiota and fecal metabolites in chickens with immunity to *E. mitis* infection.

At the phylum level, we found Firmicutes, Bacteroidetes, and Proteobacteria to be the most abundant. However, no significant difference was observed between the two groups. The Firmicutes phylum is prevalent in the intestines of poultry and involved in harvesting energy from the diet in many animals [29]. Bacteroidetes contribute to host intestinal energy metabolism through the breakdown of complex polysaccharides to produce propionic and butyric acids [30]. Proteobacteria encompass a variety of famous opportunistic pathogens, including *Escherichia coli*, *Salmonella*, and *Campylobacter*, which could have adverse effects on the health of the host [31]. Contrary to our results, the bacterial communities that were significantly increased in abundance in *E. tenella*-infected birds were Firmicutes and Proteobacteria, while Bacteroidetes was decreased [32]. We considered that this may be related to the different *Eimeria* species (*E. tenella* vs. *E. mitis*), the location of infection (cecum vs. ileum), and the infected dose (high vs. low).

At the genus level, the *Eimeria*-immunized group significantly increased the abundance of *Alloprevotella*, *Staphylococcus*, *Haemophilus*, and *Streptococcus*. Among them, *Alloprevotella* is a bacterium that produces short-chain fatty acids (SCFAs) and positively correlates with the anti-inflammatory cytokine IL-10 [33,34]. SCFAs provide energy and carbon for broilers, while also playing a role in blood flow regulation and the stimulation of intestinal cell growth [35]. However, *Staphylococcus*, *Haemophilus*, and *Streptococcus* are generally opportunistic pathogens [36,37]. Song et al. found that broilers challenged with necrotic enteritis (NE) had a significantly increased relative abundance of *Staphylococcus* in the ileum [38]. Chen et al. documented that the abundance of *Streptococcus* increased over time after *E. tenella* infection [7]. After repeated infections with a low dose of *E. mitis*, the increase in opportunistic bacterial pathogens led to gut microbiota dysbiosis. This may be related to the damage of the gut mucosal barrier and the immune response triggered by pathogen infections in the gut [39].

In contrast, the *Eimeria*-immunized group significantly decreased the levels of *Erysipelotrichaceae*, *Coprobacter*, *Bifidobacterium*, and the *Ruminococcus torques* group. *Erysipelotrichaceae* is associated with better feed conversion in broilers [40]. *Coprobacter* contributes to the maintenance of intestinal health and propionic acid production [41,42]. Earlier studies indicated that *Bifidobacterium* possesses multiple physiological functions, including modulating animals’ microflora and immunity enhancement [43]. In broilers, supplementing with *Bifidobacterium*-based probiotics increases the goblet cell cup area in the gut, significantly upregulates the expression of mucin, and increases the production of mucin glycoprotein [44]. *Ruminococcus torques* are bacteria that can produce SCFAs and are considered a dominant player in the degradation of diverse polysaccharides and fibers [45]. We found that infection with a low dose of *E. mitis* had similar effects on gut microbiota as the live vaccines currently used [46]. It has been reported that live vaccines of avian coccidiosis may have a deleterious effect on the early growth of broilers and potentially increase the susceptibility of broilers to secondary infection, although it can effectively prevent coccidiosis [47].

The gut microbiota generates multiple metabolites that are essential for regulating the intricate interactions between the gut microbiota and host physiology [48]. Metabolites derived from the intestinal tract provide valuable information for discovering potential biomarkers related to intestinal homeostasis [49]. In the present study, we found that N-undecylbenzenesulfonic acid and 1,25-dihydroxyvitamin D3 were significantly increased after chickens had been infected repeatedly with low-dose *E. mitis*. As far as we know, N-undecylbenzenesulfonic acid is an antibacterial compound against *S. aureus*, *B. subtilis*, *E. coli*, and *Klebsiella pneumoniae* [50]. The 1,25-dihydroxyvitamin D3 plays a major role in regulating immune responses, which helps the maturation of monocytes into macrophages [51]. Rajapakse et al. reported that 1,25(OH)2D3 inhibited *T. gondii* parasite growth in vivo by limiting tachyzoite invasion of cells and its proliferation [52]. After rabbits were infected with *E. magna*, a fecal metabolomics analysis also found a significant increase in 1,25(OH)2D3 metabolites [18]. Therefore, the increasing 1,25-dihydroxyvitamin D3 may help protect against *E. mitis* infection.

In addition, we noted significant reductions in trehalose, gluconic acid, 1-kestose, and ginsenoside Rg1 after the chickens had been infected repeatedly with low-dose *E. mitis*. Previous research found that trehalose improved growth and boosted immunity by moderating inflammatory cytokines in the chicken intestines [53]. Gluconic acid is generated via starch fermentation and subsequently converted into butyrate, which serves as a vital energy source for colonocytes and promotes bacterial proliferation in the host intestine [54]. Previous studies have shown that a wide variety of bacteria are capable of metabolizing glucose into gluconic acid, including various strains of the AAB genus and other genera, such as *Pseudomonas* and *Zymomonas* [55]. 1-Kestose is a fructo-oligosaccharide, produced from sucrose by the action of fructosyltransferases in plants, bacteria, yeast, and fungi [56]. One study showed that 1-kestose supplementation promoted beneficial gut microbiota in rat cecal contents and increased acetate, butyrate, and lactate levels in a dose-dependent manner [57]. A prior study demonstrated that ginsenoside Rg1 enhanced the growth and intestinal health of broilers by improving inflammatory and oxidative activities [58]. These altered metabolites further suggest that repeated low-dose *E. mitis* infections can lead to ileal microbiota dysbiosis.

## 5. Conclusions

This study demonstrated that repeated low-dose *E. mitis* infections can lead to ileal microbiota dysbiosis. This perturbation was dominated by an increased relative abundance of the pathobionts *Staphylococcus* and *Haemophilus* and an apparent decrease in the levels of nonpathogenic bacteria, including *Bifidobacterium* and *Coprobacter*. The metabolomics analysis showed that *E. mitis* infection altered 286 metabolites and 19 metabolic pathways. These findings enhanced our understanding of gut microbiota and fecal metabolic profiles of *Eimeria* spp.-infected chickens.

## Figures and Tables

**Table 1:** Output of oocysts after a total of four infections performed with low doses (2 × 10^4^) of *E. mitis* oocysts at 7-day intervals.

Infection	OPGs		
	6 dpi	7 dpi	8 dpi
1st	166,500 ± 19,200	102,000 ± 13,800	51,000 ± 7200
2nd	1500 ± 300	0	0
3rd	0	0	0
4th	0	0	0

OPGs: oocysts per gram feces; dpi: day(s) post-infection.

**Table 2:** OPG values after a high dose of *E. mitis* (2 × 10^5^) sporulated oocysts.

Group		OPG	
	Day 6	Day 7	Means
A	0	0	0 ^b^
B	306,000 ± 38,400	93,000 ± 12,600	199,500 ± 25,800 ^a^

OPG: oocysts per gram of feces; A: the *Eimeria*-immunized group was challenged with a high dose of *E. mitis*; B: the control group was challenged with a high dose of *E. mitis*. Different letters indicate a statistically significant difference.

## Data Availability

The 16S rRNA sequencing data in this study were submitted to the NCBI Sequence Read Archive (SRA) under BioProject accession number PRJNA1129660.
